# Medical Yoga Therapy

**DOI:** 10.3390/children4020012

**Published:** 2017-02-10

**Authors:** Ina Stephens

**Affiliations:** Department of Pediatrics, University of Virginia Medical Center, Charlottesville, VA 22903, USA; is8n@virginia.edu

**Keywords:** yoga, yogic practice, anxiety, depression, mindfulness, meditation, arthritis, ADHD, cardiovascular disease, inflammation

## Abstract

Medical yoga is defined as the use of yoga practices for the prevention and treatment of medical conditions. Beyond the physical elements of yoga, which are important and effective for strengthening the body, medical yoga also incorporates appropriate breathing techniques, mindfulness, and meditation in order to achieve the maximum benefits. Multiple studies have shown that yoga can positively impact the body in many ways, including helping to regulate blood glucose levels, improve musculoskeletal ailments and keeping the cardiovascular system in tune. It also has been shown to have important psychological benefits, as the practice of yoga can help to increase mental energy and positive feelings, and decrease negative feelings of aggressiveness, depression and anxiety.

## 1. Introduction

Within the past decade, yoga has infiltrated not only Western culture, but also Western medicine. The more we learn about this ancient practice, the more we realize that its benefits go far beyond increased flexibility and muscle tone. A common misunderstanding is that yoga predominantly focuses on increasing flexibility; however, although Hatha Yoga, or the physical practice of yoga, does emphasize appropriate postural alignment, musculoskeletal strength and endurance as well as balance, the study and practice of yoga incorporates mindfulness-based practices such as mindful breathing techniques, focused concentration, meditation and self-reflection.

Modern medicine has made enormous progress in controlling communicable diseases over the past century, such that it is now the non-communicable diseases (NCDs) that have reached epidemic proportions and cause the majority of deaths worldwide. The World Health Organization (WHO) estimates that 80% of NCD deaths are due to four main disease types: cardiovascular disease, cancer, diabetes, and respiratory diseases [1]. Unfortunately, lifestyle is the major causative factor in NCDs, including tobacco use, sedentary lifestyle, lack of regular exercise, unhealthy diets and chronic psychosocial stress [1,2]. Chronic inflammation and stress is a common factor of many of the NCDs, and an area where yoga has been found to be extremely beneficial. 

Recent research has shown that yogic and mindfulness-based practices can positively impact the body in many ways, including helping to regulate blood glucose levels and keeping the cardiovascular system healthy. It also has been shown to have important psychological benefits, as the practice of yoga can help to increase alertness and positive feelings, and decrease negative feelings of aggressiveness, depression and anxiety [3,4,5,6,7,8]. Some healthcare providers are responding to these positive findings—as well as the growing patient demand for an alternative approach to wellness that is natural, low-tech, relatively inexpensive and generally very safe—by incorporating medical yoga into their practices [1].

## 2. Prescription: Yoga

Medical yoga is defined as the use of yoga practices for the prevention and potential treatment of medical conditions. Beyond the physical elements of yoga, which are important and effective for strengthening the body, medical yoga also incorporates appropriate breathing techniques, mindfulness, meditation and self-reflection/study in order to achieve the maximum benefits. Medical Yoga Therapy or “Yoga Chikitsa” is the dynamic state of physical and mental ease, coupled with spiritual well-being. Yoga helps one to develop a positive state of health by not only treating illness, but also helping one to understand the underlying causes of disease. Medical yoga therapy, ideally, is an individualized, personalized and holistic approach that takes into account not only the patient’s mind, body and spirit, but also their family, support network, work situation, and culture, as part of the patient’s individualized treatment plan. As an example, if one is diagnosed with anxiety, a physician trained in medical yoga may prescribe specific breathing techniques (pranayamas), calming postures (asanas), mindfulness-based practices and/or meditation, as well as other lifestyle guidance. This type of therapy does not incur the potentially adverse effects of medications, and can produce benefits to the patient, long after their relationship with the health provider ends. 

The mindfulness and meditation aspects of yoga are ways of training the mind so that one is not distracted and caught up in its endless churning thought stream. These practices build resilience, help the patient cope with stress and manage potential triggers for anxiety. They can also promote self-reflection that may uncover the source(s) of one’s anxiety. If necessary, anti-anxiety medications and/or psychotherapy may be used in tandem; medical yoga in such cases is strongly adjunctive and complementary. 

Yoga is most powerful when it changes the patient’s general health outlook, changing the emphasis from reactive to proactive health management. The yogic definition of health or “svastha” is when the functions of the body and mind are in harmony so that they can turn inward to reach the goal of Self-realization. In yogic terms, when you are really your “Self”, you are truly at “ease”. It is the loss of the Self that creates “dis-ease”. This is a bit different than the Western concept of health, which is often defined as “the absence of disease”. In contrast, the yogic concept is that “disease is the absence of vibrant health.” Accordingly, this way of thinking reaffirms the understanding that the nature of yoga is to find one’s eternal Self of health, peace and well-being [1].

This review article will focus on: (1) The science behind medical yoga; (2) The relationship of stress to health and healing; (3) The yogic approach to health care; and (4) The research behind medical yoga therapy.

## 3. What Is Yoga

Yoga is not a particular denomination or religion, but an age-old practice based on a harmonizing system for the body, mind, and spirit to attain inner peace and liberation [9,10]. Yoga is a tool that can deepen and benefit anyone, of any religion. It does not conflict with personal beliefs; it is simply a vehicle to help one transform oneself by promoting conscious connection with oneself, the world, and the highest truth. There are many traditional paths of yoga, including tantra, mantra, kundalini, bhakti, jnana, karma, raja yoga, and others, all of which have their own techniques to awaken these connections. According to the classic text of the *Yoga Sutras of Patanjali*, “yoga” is the complete “inhibition of the modifications of the mind” [9] or quieting of the constant chatter in one’s mind so that our True Selves can manifest, rest in our own true nature and be free of suffering. Disease, as described in the sutras, is said to be an impediment to spiritual practice, growth and freedom from suffering [9]. Traditional yogic practices include breath control and techniques (pranayama), meditation (including mindfulness), the adoption of specific bodily postures (asanas) and self-reflection (scriptural or self-study) [9,10].

As a mindfulness practice, yoga requires one to be fully aware in the present moment. This practice helps to diffuse anxiety (which largely concerns the future), and sadness (which largely concerns the past). All the yogic practices use present moment, non-judgmental awareness as the foundation. Of course, such presence benefits the healthcare provider as much as the patient: if one can learn to pay attention to oneself, one can really pay attention to one’s patients. One can then teach the patient to pay attention to him or herself as well.

## 4. Medical Yoga Prescription

Medical yoga as considered here comprises the use of traditional yogic practices to prevent, cure, and/or ameliorate disease. The ideal medical yoga prescription includes the yogic practices of breathing techniques, bodily postures, meditation techniques and self-reflection; a healthy, nourishing diet; reducing substances such as caffeine, tobacco, drugs and alcohol; healthy sleep hygiene and appropriate support, which may include family, spouse, children, friends and/or support groups, with or without psychotherapy. It is important that medical yoga therapy should start gently and with self-compassion.

For providers considering adding yoga to their therapeutic armamentarium, the best place to start is to consider yoga therapy as a complement to their patient’s current medical treatment. Yoga alone should not be considered a substitute for appropriate medication or psychotherapy. However, in situations where a patient is at risk of an illness but does not currently need more intensive therapy, introduction of yogic practices may forestall or prevent progression to the point where medical therapy is needed. Patients whose daily activities produce back strain or who have inherently stressful lives may benefit from yoga as a prophylactic strategy, thereby potentially avoiding more intensive interventions. It is also important to remember that not all yoga is appropriate for all patients and that yoga therapy is different than simply taking a group yoga class where the yoga instructor may not be aware of an individual student’s health concerns or problems. Most certified yoga teachers, or instructors, have received some training in anatomy and physiology; however, this training can be quite varied and is not equivalent to the training required by the yoga therapist or healthcare practitioner. As noted previously, yoga therapy, different from a yoga class, starts with a detailed history and physical examination and assessment from the health practitioner. 

Yoga brings the autonomic nervous system into healthy balance by stimulating the parasympathetic nervous system [11,12]. The sympathetic nervous system, or our “emergency response system,” is activated when our body or mind feels threatened or perceives being stressed, whether that be a “positive” or a “negative” stress. This “flight or fight” response results in vasoconstriction, causing decreased blood flow to the extremities and the digestive system in order to prepare one for survival [11]. One’s heart rate and blood pressure increase, the liver converts glycogen to glucose and releases glucose into the bloodstream, the bronchioles dilate, and the blood flow pattern changes, leading to decreased digestive system activity and reduced urine output. In contrast, the parasympathetic system is stimulated when one relaxes; it is often called the “rest and digest” mechanism of our nervous system. The parasympathetic system stimulates blood flow to the digestive system, brain, extremities and sexual organs [11]. As many of us go through our day, what is happening on the “outside”—i.e., what we may think or what we may encounter—causes a constant interaction between the two facets of the nervous system. Yogic practices work by decreasing physiologic arousal and quieting down this continual play of the autonomic system. They can reduce one's heart rate and blood pressure, ease one’s respirations and increase heart rate variability—all signs of improved parasympathetic tone [13].

## 5. Stress

Many in today’s society live in “sympathetic overdrive.” This occurs when the body perceives a continuous stressor, and the short-term sympathetic stress response persists all day long [11]. Unfortunately, our bodies were not really set up in a way to handle these continual stressors [12]. Due to the continued excitation, one of the major neuroendocrine systems of the body, the hypothalamic-pituitary-adrenal (HPA) axis is set in motion. The HPA axis regulates and helps to control many different bodily processes including one’s reaction to stress, immune function, digestion and energy expenditure and storage. The axis is set in motion with release of corticotropin-releasing factor (CRF) from the hypothalamus. CRF then stimulates the anterior pituitary gland to release adrenocorticotropic hormone (ACTH), which subsequently stimulates the adrenal cortex to produce and release cortisol [14]. In response to the stress, cortisol functions by aiding in the metabolism of fat, protein, and carbohydrates by converting them to glucose through gluconeogenesis. This longer-term stress response subsequently raises blood glucose levels, suppresses the immune system, and causes retention of sodium and water by the kidneys, with subsequent increased blood volume and blood pressure. At a certain blood concentration, cortisol will exert a negative feedback to the hypothalamus and the pituitary gland with decreased discharging of both CRF and ACTH. At this point, homeostasis returns to the body and entire system. If, however, the body continues to be in a state of stress, real or perceived, this cycle perpetuates with subsequent sustained HPA axis activation [14,15]. 

Not all stress has unfavorable effects, of course. When the body is able to tolerate the stress, and can use it to boost one’s performance, the positive effects are numerous and impressive. There are over >1400 biochemical reactions that the body has in response to stress, with subsequent effects on one’s body, mind, emotions and behavior [12]. However, stress becomes negative when it exceeds one’s ability to cope. At this point, the body systems can become fatigued, causing multiple problems, both physically and emotionally [15,16]. 

Chronic stress is experienced when there is a mismatch between a perceived demand and one's ability to cope with that demand; it includes one’s emotional and mental response to the outside situation. When one is continually stressed, whether that stress is real or perceived, his or her nervous system can be shifted towards the state of sympathetic overdrive [11,12]. At this point, the actual activity of the sympathetic nervous system decreases with a decline in the release of epinephrine, but corticosteroid release continues to be activated at above-normal levels. In such a case, the individual may no longer even recognize that they are in a stressful state. Moreover, chronic stress can be precipitated by any number of factors and conditions, some regular and unavoidable: constant worry, daily life hassles, frustrations over one’s job or family, traffic jams, finances, poor sleep or eating habits, demands on time, and challenging life situations all create stress [12]. Stress can even also be triggered by one’s memory of difficult or scary past situations that have previously occurred. This state of high stress causing continuous sympathetic stimulation is epidemic in our society, from the corporate executive down to the young child on a busy school and activity schedule.

So stress is part of our lives—does it matter? In fact, the burden due to stress-related illness is quite concerning. The Centers for Disease Control and Prevention estimates that stress accounts for about 75% of all physician visits [12,16,17], and up to 80% of all visits to primary care providers are for stress-related complaints [12,17]. These involve a wide spectrum of complaints, including headache, back pain, hypertension, arrhythmias, irritable bowel syndrome, insomnia, depression, anxiety, skin problems, fatigue, obesity, migraines, hyperlipidemia and accidents.

Research has shown that continued and chronic stress can result in a generalized immunosuppressive effect that prevents the body to initiate a timely and appropriate immune reaction [16,18,19,20]. During acute stress, norepinephrine, epinephrine and ACTH are released, signaling certain kinds of cells to become mobilized into the bloodstream, potentially preparing the body for the “fight or flight” response [1]. Acute stress increases levels of pro-inflammatory cytokines in the bloodstream [21]. Whereas inflammation is a necessary short-term response for eliminating pathogens and initiating healing, chronic systemic inflammation causes dysregulation of the immune system and increases one’s risk for chronic diseases [20,21,22]. Chronic stress, like acute stress, is associated with high levels of pro-inflammatory cytokines, but with potentially different health consequences [22]. Type 1 cytokine protective immune responses have been shown to be suppressed during times when the body is undergoing challenges of chronic stress, while pro-inflammatory and type-2 cytokine responses are activated [23,24]. Chronic or long-term stress can also suppress immunity by decreasing the number of immune cells as well as their function. These phenomenon may exacerbate pro-inflammatory diseases and thus potentially increase one’s susceptibility to infections and possibly different types of cancer [24]. 

Chronically high levels of cortisol can also be neurotoxic [14]. These high levels have been associated with accelerated aging, cognitive inhibition, impaired memory and the ability to learn, increased anxiety and fear, as well as depression and anhedonia [25,26,27]. Areas of the brain, including the hippocampus and the prefrontal cortex (PFC), both with high cortisol receptors, can become impaired with high cortisol levels. Prolonged stress, both physiological and psychological, with continued high levels of blood cortisol can induce lowered metabolic rates and decreased synaptic densities in the hippocampus and the PFC [25,28,29,30]. Even perceived stress has been shown to negatively correlate with overall PFC volume, specifically in white matter volume of the PFC, specifically in the ventrolateral and dorsolateral PFC [31].

Cortisol activates the amygdala, the body’s “threat center.” The amygdala is an almond-shaped brain structure that is crucial in the stress response. An enlarged and hyperactive amygdala is often observed during stressful conditions placed on the body [14,32,33,34,35]. Of interest, studies performed on decreasing stress revealed that following stress-reduction interventions, where patients reported significantly reduced perceived stress, decreases basolateral amygdala gray matter density, as seen on Magnetic Resonance Imaging (MRI) scans [32,36]. 

Stress, of all kinds, is thought to play a significant role in the development of depression by depleting the body’s “positive” neurotransmitters, such as gamma-aminobutyric acid (GABA), serotonin and dehydroepiandrosterone (DHEA) [37,38,39,40,41,42]. Dysregulation of the HPA axis is often seen and reported in those with depressive disorders. In addition, both the dopaminergic and serotonergic systems are thought to be involved in the development of depression if they become dysfunctional or dysregulated [43]. Studies done in rats have shown that chronic stress can induce a depressive state, along with notable dysregulation of both their HPA axes and their dopaminergic and serotonergic systems in the PFC. Release of GABA has also shown to be impaired in these rats as well [40,43]. Other mood disorders, including anxiety, are also thought to be aggravated by stress-induced modifications of the PFC. Of note, stress may impact the prefrontal GABA pathways. This can impair the function of the limbic structures, which are known to control one’s emotions and behavior [44,45]. 

## 6. How Do Yogic Practices Work?

Yoga-based practices of postures (asanas) and movement sequences are usually taught in conjunction with some type of breathing and/or meditation technique [1]. This type of mindful movement with slow, rhythmic breathing is more likely to promote parasympathetic and vagal tone compared to other forms of exercise [1,11,46,47]. Improved vagal tone is reflected by increased heart rate variability (HRV), which is the variation in the time interval between heartbeats. This physiologic phenomenon can be predictive of how readily the heart rate returns to normal, or quiets down, after increasing in response to a stressor. Decreased HRV is associated with poorer myocardial function, often seen after a myocardial infarction, and is seen with increased sympathetic activity. Increased HRV with high frequency activity is associated with increased parasympathetic activity. Yogic breathing techniques—in particular, alternate nostril breathing, which involves breathing through the left and right nostril alternately—has been associated with increased parasympathetic activity, increased HRV and decreased systolic blood pressure [48]. Slow and rhythmic breathing has also been shown to promote the release of prolactin and the hormone oxytocin, which can foster feelings of friendship, calmness and bonding to others (released during childbirth which may help the mother relax and bond with her newborn during a very painful process) [1,49]. These yoga practices also reduce circulating levels of cortisol and have been demonstrated to reduce the manifestations of stress. With practice, there is decreased firing from the locus coeruleus, which is the principal site in the brain for synthesis of norepinephrine in response to stress and panic. This decreased norepinephrine output helps the body to relax and quiet down with reduced respiratory rates and heart rates. The decreased sympathetic output decreases the release of corticotropin releasing factor, with resultant decrease in cortisol output [50,51,52].

An open-label study from 2013 performed on a cohort of 54 outpatients with clinical depression at a tertiary care psychiatric hospital revealed that, as a group, this cohort had higher levels of serum cortisol compared to healthy controls [50]. These 54 subjects were offered yoga classes as adjunctive therapy for their depressive symptoms. The yoga module was taught to the group over a month’s time and subjects were advised to practice daily at home. Antidepressant medication was prescribed in addition if advised by the subject’s psychiatrist. The cohort was broken down into three groups: Group (G) 1 received conventional drug therapy (DT) alone (N-16), G2 received yoga therapy (YT) alone (N-19), and G3 received DT along with YT (N-19). A G4 group was used as healthy controls (N-18). The Hamilton Depression Rating Scale (HDRS) was used to rate subject’s depressive symptoms, along with measurements of serum cortisol, obtained both at baseline and after three months of intervention. In the total sample, with good adherence, and irrespective of treatment method, the cortisol levels were found to be decreased significantly at the end of treatment in all subjects. Of interest, however, is that more subjects in the yoga groups (both YT and YT plus DT) had significantly greater drops in their cortisol levels as compared to the drug-only (DT) group (*p* < 0.008). In the YT-only group, there was also a high correlation between decreased cortisol levels and lower scores on the HDRS surveys, indicating a positive antidepressant effect as well (*p* < 0.001) [50].

## 7. Meditation

It appears that formal meditation practice can change both brain structure and function [53]. It has been found that people who do more meditation practice develop more robust brain structures in certain areas. Multiple studies have shown that yogic practices such as mindful meditation can increase both cortical thickness and gray matter, particularly in areas controlling emotional regulation and executive functioning. These regions notably include the insula, the ventromedial pre-frontal cortex and anterior cingulate cortex (ACC) [53,54]. The insula is involved with proprioception, self-awareness and emotional regulation. The ventromedial PFC is the brain’s center for executive functioning, including planning, problem solving and emotional regulation. The ACC is the self-regulatory process center, giving one the ability to monitor attention conflicts and allow for more cognitive flexibility [55,56,57,58].

Meditation has been shown to increase the thickness of the left hippocampus, the region of the brain that functions in the formation of long-term memory, emotional regulation and cognition, as well as being a critical area of the brain that plays a vital role in the resiliency to chronic stress and depressive states, possibly due to expression of hippocampal neurotrophic protein (brain-derived neurotropic factor or BDNF) [59]. Resiliency to stress, stress-related depression and post-traumatic stress are housed in the hippocampus—multiple studies have shown that increased hippocampal activation correlated negatively with post-traumatic stress disorder (PTSD) and depression symptoms, and positively with resilience [25,55,60,61,62]. PTSD patients show considerable reduction in volume of the hippocampus, decreased ventromedial PFC activity and insufficient inhibition of the amygdala, all resulting in increased fear, persistent negative emotions, impulsivity, anxiety and depressive rumination. 

Mindfulness meditation, particularly in long-term expert meditators, has been shown to diminish activity and size in anxiety-related areas of the brain such as the amygdala, as well as to increase the size of the PFC and insular cortex, both controlling emotional regulation. These meditators have also been shown to have diminished functional connections between the amygdala and the PFC, allowing for less reactivity to stressors [55,63,64]. Meditation also appears to be effective for PTSD and depression symptoms, although more high-quality studies are needed [65,66]. 

A recent study suggested that hatha yoga postures (asanas) alone can also improve stress and increase relaxation dispositions. This randomized controlled trial performed in 2015 among college students with high psychological stress assessed the effects of sun salutations or suryanamaskar (defined as a series of 12 physical postures) on relaxation dispositions (R-dispositions). Suryanamaskar is a traditional asana sequence that is a well-known yogic exercise; however, there are few studies on the physiologic effects of regular suryanamaskar practice [67,68]. In this study, the intervention group was to practice a daily suryanamaskar session for 14 days, which was composed of a short warm up, 13 rounds of suryanamaskar with mantras and breathing, followed by a cool down in a sitting position. The set of exercises took approximately 20 min to complete daily. No specific activity was given for the control arm to perform. After 14 days, the experimental group scored higher on multiple aspects on the surveys with increased points compared to the control group for: physical relaxation, mental calmness, feeling at ease and peace, being more well-rested and refreshed, improved strength, awareness and joy. In addition, the intervention group scored lower on fatigue, somatic stress, worry, and negative emotional feelings when compared with the control group [69].

## 8. Yoga and Neurotransmitters

Yoga practices can increase multiple neurotransmitters and hormones such as GABA, serotonin, and dopamine—all natural anti-depressants [11]. They have been shown to increase levels of melatonin, helping to initiate sleep, improving sleep quality and sleep regulation, as well as increasing levels of oxytocin, the “bonding hormone”, thus helping with feelings of connectedness and “being seen and heard” [70,71,72,73,74].

GABA is one of the body’s chief inhibitory neurotransmitters, working to reduce neuronal excitability and activity throughout the central nervous system (CNS). GABA acts as an important player in the body’s response to stress, fear, depression, anxiety and sleep regulation. Lower-than-normal levels of GABA in the brain have been associated with schizophrenia, depression, anxiety, post-traumatic stress disorder, epilepsy and sleep disorders [73].

Multiple kinds of both anxiolytic and anti-depressant medications work by increasing GABA levels in the central nervous system. As mentioned earlier, yoga practices seem to be effective by bringing the parasympathetic and sympathetic systems into balance (often by increasing parasympathetic tone and decreasing sympathetic firing), and does this in large part by increasing GABA activity [75]. Meditation increases activity in the PFC as well as stimulates the thalamus (in particular, the reticular nucleus), which increases production and delivery of GABA throughout the CNS. Studies have shown that GABAergic inhibitory interneurons result in cortical inhibition which has been implicated in improved cognitive performance and enhanced emotional regulation capabilities [76]. Multiple studies have shown that the practice of yoga and meditation may work as well as other therapies in increasing GABA levels in the brain [77,78,79].

## 9. Yoga and Telomeres

As meditation, mindfulness practices and yoga have been moving more and more into the mainstream, it is becoming more apparent that these practices may work to keep our minds and bodies from withering with age by potentially stabilizing, and even lengthening telomeres. Telomeres are small, repetitive, chromosomal sequences found at the end of chromosomes which protect the chromosome from deterioration and cell death. They keep the chromosome stable. Telomere shortening, or unraveling, affects how quickly cells age. As they shorten, the chromosome’s structural integrity weakens. Telomere length has been found to be a prognostic marker of aging, disease and premature morbidity in humans. Shorter telomeres are associated with cell aging, cell death, premature aging and a broad range of aging-related diseases, including cardiovascular disease, cancer, stroke, dementia, obesity, osteoporosis, Alzheimer’s, macular degeneration, acquired immunodeficiency syndrome (AIDS), and osteoarthritis. They have also been associated with pediatric syndromes such as Progeria, Cri-du-chat, Down’s syndrome, tuberous sclerosis, dyskeratosis congenita and Fanconi anemia. Telomere shortening is prevented by the enzyme telomerase. Of note, chronic stress may potentially speed up the aging process through decreased telomerase activity and telomere shortening [80]. 

In 2008, Dean Ornish found a significant association between comprehensive lifestyle changes (including yoga, meditation, breathing, stress management and a healthy whole-food, plant-based diet), and increased telomerase activity in human peripheral blood mononuclear cells. Of interest, the participants of this study also showed significant reductions in psychological distress and low-density lipoprotein cholesterol [81]. In 2013, a five-year follow-up study was completed on the same participants and controls. The participants were found to have persistently increased telomere length and telomerase activity over their age-matched controls. The study also compared blood samples from 2008 and 2013, evaluating both relative telomere length (RTL) and telomerase enzymatic activity per cell. Of interest, after five years, RTL was found to have increased in the lifestyle intervention group and to have decreased in the control group with the difference between the two groups as statistically significant [82]. This was the first controlled trial to show that any lifestyle intervention might lengthen telomeres over time.

Meditation has also been shown to be potentially protective regarding RTL. An intriguing study from 2013 evaluated a group of 15 subjects practicing Loving-Kindness Meditation (LKM) compared to a control group of non-meditators (n-22). LKM, or *Metta bhavana* from the Buddhist tradition, is a method of developing compassion by directing well-wishes towards others. It can be practiced by anyone, regardless of religious affiliation. It is a meditation of care, concern, friendship and compassion for oneself and others. Peripheral blood leukocytes were then measured for RTL in both groups. The LKM practitioners were found to have significantly longer RTLs than the control group, particularly for the women (*p* = 0.007). Although this study was limited by its small sample size, these results are exciting as they suggest that meditation—in particular, LKM practice—might increase RTL, a biomarker associated with aging and longevity [83].

## 10. Yoga and Inflammation

As noted above, inflammation is the body’s natural immune response to infection, injury, and stress. However, inflammation can have serious health implications when it becomes prolonged and chronic. Chronic systemic inflammation may not be as apparent as acute inflammation, and can persist undetected at low levels for years. This can slowly damage the body, lead to the development of chronic diseases and increase one’s risk for type II diabetes, atherosclerosis, cardiovascular disease, autoimmune disease and age-related diseases. 

As previously discussed, yoga is beneficial for decreasing both acute and chronic stress levels. In multiple studies, yoga has been found to decrease inflammatory markers such as C-reactive protein and other inflammatory cytokines in the blood, while increasing levels of multiple immunoglobulins and natural killer cells [11,84,85,86]. Recent research has also shown that those who practice yoga regularly have higher levels of leptin and adiponectin in their bodies, both natural chemicals that work to alleviate inflammation in the body. Adiponectin has been found to be a key component of endothelial function and is cardioprotective [1,87,88]. 

A very interesting recent discovery is that even a small amount of practice may make a significant difference. A notable study by Yadav et al. in 2012 looked at how even a very brief (10-day) yoga-based lifestyle intervention can reduce the inflammatory markers of stress in patients with chronic diseases. In this trial, 86 patients (44 female/42 male) with chronic inflammatory diseases, including those who were overweight/obese received a ten-day yoga-based intervention program, including yoga asanas, pranayama and both individual and group stress management sessions. Statistically significant changes (*p* < 0.05) were seen after this short ten-day intervention with decreased cortisol, tumor necrosis factor-alpha (TNF-α) and interleukin-6 (IL-6) levels, and elevated levels of β-endorphins [89].

Another randomized, controlled clinical trial from 2014 also showed that practicing yoga for as little as three months can not only lower markers of inflammation, but can also make big differences in symptoms of fatigue in breast cancer survivors [90]. In this trial, 200 women were recruited and randomly assigned to either 12 weeks of 90-min hatha yoga classes twice per week, or a wait-list control. Breast cancer treatments were fully completed before the onset of the study.

Multiple inflammatory markers were evaluated as part of the outcome of this trial as well as multiple different surveys assessing both fatigue and depression. At three months post-treatment, comparing the yoga group to the control group, fatigue was found to be lower as well as a number of inflammatory markers such as IL-6, TNF-α and IL-1 β and scores for vitality were found to be higher (all statistically significant with *p* < 0.05) [90].

Of particular interest, this study also showed that the more yogic practices that the women did, the better their outcomes. Also significant was that the results were sustained: after six months post-intervention, both fatigue and inflammation were still lower in the intervention group as compared to controls (decreased by 57% and 20%, respectively) [90]. This suggests that yogic practices can continue to make significant sustained differences in signs and symptoms of inflammation even months later. 

## 11. Yoga and Back Pain/Arthritis

The American College of Rheumatology states that exercise and physical activity is a necessary part of an effective treatment program for patients with both osteoarthritis and rheumatoid arthritis [91,92]. In these patients, exercise has been shown to have a vital role in promoting joint health without worsening disease. Patients suffering from arthritis who exercise regularly have less joint pain, more vitality, better sleep, reduced morning joint stiffness and improved daily living function. In particular, yoga incorporates important elements of body awareness such as proprioception, coordination, balance and postural alignment, all of which are particularly important in individuals with joint disease [93].

A comprehensive review of randomized controlled trials that evaluated yoga as an intervention for chronic low back pain (CLBP) supported the practices and found them to be efficacious on short-term improvements in functional disability [94]. In fact, yoga therapy has been shown to improve pain, back function, spinal mobility, depression and anxiety in patients with CLBP to a greater degree than physical therapy [95,96]. 

The largest, most rigorously conducted randomized controlled trial (RCT) of yoga and arthritis to date was published in 2015 by Moonaz et al. at Johns Hopkins University. Most importantly, this trial examined the safety, efficacy and feasibility of yoga for sedentary patients with both rheumatoid and osteoarthritis, with significant outcomes revealing important physical and mental health benefits in these individuals with regular yoga practice. After eight weeks of intervention, improvements were seen in physical pain, general health, vitality, ability to carry out Activities of Daily Living (ADLs), balance, upper body strength and mental health scales (*p* < 0.05). Of great interest, almost all benefits that were seen were still observable nine months after study completion [97].

## 12. Yoga and Cardiac Disease

Cardiovascular disease (CVD) encompasses a broad spectrum of syndromes, including atherosclerosis, stroke, arrhythmia, hypertension, hyperlipidemia, heart disease and peripheral vascular disease, and is the leading cause of mortality, morbidity and disability worldwide [1]. Although there have been tremendous advancements in medications, treatment plans and programs for both the prevention and treatment of CVD, there are still a number of challenges in implementation of these programs, and limitations of the treatments. Multiple risk factors are known to cause oxidative stress, leading to endothelial disruption and dysfunction. These include dyslipidemia, diabetes, hypertension, obesity, smoking and psychological stress, which can in turn start a cascade of events involving inflammatory and vasoactive mediators, in particular, interleukin-6, fibrinogen, C-reactive protein and tumor necrosis factor-alpha, that lead to the development of CVD [1]. Yoga therapy may be a significant and cost-effective therapy for CVD by interrupting a number of these different events along this cascade [1,11,88,98,99,100].

Of interest is a study done by Sarvottam et al. suggesting that even a short-term yoga-based program may reduce the risk for CVD. In this trial, a ten-day yoga intervention program was found to significantly reduce the body mass indices and systolic blood pressures in 51 overweight and obese men. These men were also found to have significant changes in certain inflammatory markers with decreases in IL-6 and elevation of adiponectin [88]. 

According to the American Heart Association, yoga practices can help to lower blood pressure, increase lung capacity, improve respiratory function and heart rate, improve circulation and boost muscle tone [101]. As previously discussed, yoga can reduce stress both by balancing the autonomic nervous system with increased parasympathetic and reduced sympathetic activities, respectively, thus optimizing and restoring the body’s homeostasis (decreasing allostatic load), as well as decreasing the reactivity of the HPA axis. By decreasing both of these pathways, yoga can interrupt multiple different inflammatory events on the cascade toward CVD and enhance cardiovagal function [102]. 

An intriguing study by Krishna et al. in 2014 evaluated the effects of yoga therapy in patients with heart failure. Parameters including heart rate, blood pressure, heart rate variability (HRV) and rate pressure product (RPP) were measured before and after yoga intervention. As mentioned above, increased HRV is predictive of how readily the heart rate returns to normal, or quiets down, after increasing in response to a stressor—a sign of parasympathetic tone—and RRP is an index of myocardial O_2_ consumption and load on the heart. Both of these are measures of cardiac autonomic function. In this trial of 130 heart failure patients, randomly assigned to receive either a yoga intervention program or standard medical therapy alone, heart rate (HR), blood pressure (BP), and measures of cardiac autonomic function were assessed before and after the 12-week intervention. The results were quite significant with *p* values all <0.05 regarding decreases in HR, BP and RPP in the yoga group compared to the controls. Sympathetic nervous system modulation (measured by Lfnu—low-frequency normalized unit) decreased significantly and parasympathetic nervous system modulation (measured by Hfnu—high frequency normalized unit) increased significantly as well in the intervention compared to the controls—both signs indicating improved HRV [103].

This study suggests that there could be a great benefit of yoga therapy as an adjunct to medical treatment in patients with heart failure. In addition, because it is important to consider that in patients with severe and/or decompensated heart failure physical exercise may not be well tolerated, yoga, particularly gentle asanas, breathing exercises and meditation, may be easily tolerated by these individuals [1].

As a complementary and integrative therapy, yoga for the management of hypertension has been studied in numerous randomized controlled trials. On average, the overall effect of yoga therapy results in a reduction of systolic BP of approximately 10 mmHg and approximately an 8 mmHg reduction in diastolic BP [75,104]. Of note, yoga seems to be efficacious only for hypertension, not for pre-hypertension. It is also important to recognize that at this time yoga therapy can only be recommended as an adjunct to antihypertensive pharmacological treatment, not as an alternative therapy alone. Breathing and meditation seem to be the important components of the yoga interventions as well rather than physical yoga asanas for hypertensive patients. These are the components of the yogic practice that can increase parasympathetic activity and decrease sympathetic tone, which counteracts the surplus of sympathetic activity associated with hypertension. In addition, the specific components of yoga practice may help one to self-regulate, so that the mind and body can work to bring one’s physical, emotional, autonomic and psychological systems into balance, which is most critical when the body is under stress [105]. An interesting study conducted in 2013 on the effects of Iyengar yoga supports this theory. After an eight-week yoga program intervention, outcomes from surveys strongly suggested that the yoga practice helped to benefit self-regulation in terms of physical function, enriched sleep quality, dietary improvements with improved lifestyle choices, reduction of stress and anxiety and enhanced calm mental/emotional states in the study participants [106]. Despite the increasing evidence that yogic practices may reduce blood pressure, it is important to recognize that many of the studies done have also included diet modifications, exercise and/or supportive guidance and counseling—all part of the “yogic lifestyle”. The exact mechanisms as to the potential benefits of yoga in controlling blood pressure remain unknown at this time. Additional rigorously controlled trials are needed to further investigate the potential benefits of yoga for improving blood pressure in those individuals with both pre-hypertension and hypertension to help determine optimal yogic practices, yoga program design and treatment plan [1,75,105,107].

Yoga therapy may also be quite useful as a complementary therapy for atrial fibrillation (AF). It is well known that the autonomic nervous system plays a pivotal role in the pathophysiology of AF, and it has been proposed that an imbalance in both the sympathetic and parasympathetic nervous systems contribute to the disease entity [108,109]. In a trial performed in 2013 by Lakkireddy et al. the impact of a three-month yoga intervention program was evaluated in 49 patients with paroxysmal AF. Both symptomatic and asymptomatic AF episodes were assessed for a three-month control period prior to the intervention, and during the intervention. Statistically significant decreases in both symptomatic and asymptomatic AF episodes were observed with *p* values < 0.001. Of interest, feelings of both depression and anxiety were also found to be reduced significantly in study subjects as well as improvements on several measures of quality of life including general health, physical functioning, vitality, social functioning and mental health [108]. The benefits of yoga therapy in these patients with AF are thought to be due to restoration of sympathetic and parasympathetic balance at the level of HPA axis as well as decreasing both inflammation and oxidative stress, resulting in the suppression of atrial remodeling, micro-reentry circuits and triggers for AF; however, the exact mechanism remains unknown [1,108].

## 13. Yoga for Pediatrics

A growing amount of evidence is showing that yoga and other mindfulness-based practices are paramount for today’s children. Due to various new demands and standards in today’s society, children and adolescents experience stress and mental health challenges that have not been seen in generations past. In a society exploding with technology, children now are confronted with many daily distractions and temptations, with resultant overstimulation and pressures from their peers. There are more stresses on families with reduced downtime and quiet time caused by the overscheduling of activities, overvaluing productive time and greater pressure to succeed academically. Recent research shows that the current generation of young adults is the most “stressed-out” generation, compared with their predecessors [110]. There is constant stimulation through technology, internet and social media, as well as extensive media usage by children and adolescents in today’s world. Not only are children and young adults under more stress, but they also have fewer coping skills to manage these stressors. As with adults, when children internalize stress, it is often manifested physically, resulting in health issues such as insomnia, chronic abdominal pain, headaches, depression, anxiety and mood swings [111,103]. For the past number of years, schools have been cutting programs such as life skills courses and physical education classes. When these stress management skills are not learned at an early age, it only becomes harder to learn them as the children get older. Yoga may help children, adolescents and young adults cope with stress by teaching them self-regulation skills to control emotions and stress at a young age. These practices would, in turn, help their well-being and mental health, improve overall resiliency and help to positively keep their lives in balance. Yogic practices help the body to connect to the mind by helping one to focus on the present moment and clearing the mind of overwhelming thoughts. Even very young children can learn to benefit from yogic breathing techniques, which can help to calm and distract toddlers from a temper tantrum or help them to sleep. According to the National Institutes of Health, children who practice yoga have an increased sense of self-awareness and self-confidence. Concentration skills are enhanced [112,104]. These learned mind-body skills can also help a child reexamine a difficult, or even painful, experience into one that bolsters their sense of resiliency [113,105]. This, in turn, may contribute to improved attention, self-esteem, empowerment and good mental health [114,115,116].

According to the 2007 National Health Interview Survey (NHIS), mind-body therapies, including yoga, were the most favored complementary and alternative medicine (CAM) practices among children with behavioral, emotional or mental health problems. Per the 2012 NHIS, the use of yoga and yoga therapy in children had increased since 2007 from 2.5% to 3.2% (more than 400,000 children) [113]. Of note, older children between the ages of 13 and 17 were noted to use these mind-body therapies more often, and were more commonly used by female patients versus male patients (5.7% vs. 1.7%) [117]. Of interest, a survey taken in 2007 showed that in children and adolescents with chronic pain, over 60% of the subjects tried at least one CAM approach for pain. Of these practices, yogic practices and therapies were used by 32% as their first choice of CAM therapy [118].

Compared to research performed in adults, there is unfortunately a lack of good RCTs on the safety and efficacy of yoga among the pediatric population. In 2008, Galantino et al. performed a systematic review and found 24 studies of yoga for children which included case-control studies, pilot studies, cohort studies and RCTs. Although this review concluded that there was certainly positive evidence regarding the use of yoga in the pediatric population, more research is imperative [119,120]. Since then, there have been no recent systematic reviews of pediatric therapeutic yoga [114]; however, a growing number of RCTs are being performed. In a recent bibliometric analysis of yoga studies published between 1975 and 2014, there were 366 studies found with 31 of the studies (9.9%) including children [121]. In 2010, Kaley-Isley et al. performed a comprehensive review, and subsequently summarized applicable studies with children and adolescents treated with yoga therapy practices as an intervention. The majority of these studies showed benefit as well as very few adverse effects. Among these studies, the areas for which yoga was used for therapy in children include physical fitness, cardiac and respiratory symptoms, mental and behavioral health, developmental syndromes, irritable bowel syndrome, eating disorders including obesity, anorexia and bulimia, cancer and prenatal effects on birth outcomes [1]. In 2016, 14 individual controlled studies were evaluated, showing that yoga, as a CAM treatment and approach, appears to be an encouraging therapy and stress management intervention for children and adolescents, again with a very low rate of reported adverse effects. Yoga therapy can have significantly favorable effects on psychological and cognitive functioning, particularly in patients with emotional, mental and behavioral disorders [117]. Of note, many of the studies reviewed had methodological limitations, small sample sizes, lack of randomization and much variability between yoga practice intervention methods so that clear conclusions were not possible, and it was suggested that to obtain the most benefits from therapeutic yoga in children, more regulated research efforts are warranted [117,122].

There has been increasing interest in the use of mind-body techniques and therapies for children and adolescents with focusing, concentration and attention disorders. If yoga and mindfulness helps one to focus inward and pay attention, it would be only natural to assume that these types of therapies would be of great benefit to those who have difficulty with inattentiveness. Ideally, this increased focus would potentially increase attention naturally, even in children with challenging attention disorders. 

In 2012, a systematic review of 124 trials examining the evidence for efficacy of yoga in the treatment of a number of different pediatric psychiatric disorders, of which 16 met appropriate criteria for the final review, showed emerging evidence (Grade B) to support a role for yoga in treating children with Attention Deficit and Hyperactivity Disorder (ADHD) in two RCTs [123]. Among these two RCTs was an intriguing randomized trial from 2004, published in the *Journal of Attention Disorders*, which showed that boys diagnosed with ADHD, on appropriate medication, reduced their symptoms in inattentiveness and behavior when practicing yoga regularly. In this trial, 16 boys with ADHD were randomized to receive either yoga therapy (YG) or cooperative activities (CG) in addition to their previously prescribed medications for a total of 20 weeks. The YG received one hour of hatha yoga per week along with breathing and relaxation techniques. Assessments were performed both pre- and post-intervention using the Conners’ Parent and Teacher Rating Scales (CPRS and CTRS). Although there was improvement in both groups, the YG group showed significant improvement on five subscales of the CPRS. Positive changes on the CTRS were also seen in those who attended more yoga sessions and who engaged in more home practice. Although this was a small study, the trial did suggest that yoga may be a useful CAM therapy for adjunctive management, along with medical management, in children with ADHD. Larger and more collaborative studies on yoga’s potential benefits for these children are needed [124].

Another trial from 2006 by Haffner et al. evaluated 19 children with ADHD and randomized them to receive either YG or conventional motor training (CG), along with their current medication regimens. The YG received 2 h of hatha yoga weekly for a period of 34 weeks. Outcome measures here included test scores on a task requiring focused attention, as well as both parent and teacher ratings of ADHD symptoms. Results from this trial revealed that the yoga training was superior to the conventional motor training. This pilot study also demonstrated that yoga can be an impressive adjunctive treatment for ADHD, though again limited by its small sample size. These researchers advocate for further research into the impact of yoga on children with ADHD [125].

What about the possibilities of yoga entering the school curriculum? It has become increasingly commonplace for large companies and offices to incorporate yoga and meditation facilities for their employees as a means to help improve concentration, refresh focus, improve motivation and counteract prolonged sitting at a desk orworkbench all work-day long. Schoolchildren, who also spend hours working and sitting all day, may benefit to the same degree. 

There have been a few RCTs have evaluated the effects yoga within a school curriculum particularly in the prevention of mental health problems and enhancement of psychosocial well-being. One RCT from 2012 by Khalsa et al. studied 109 students randomly assigned to receive either an 11-week yoga education program versus regular physical education. In this trial, students were asked to self-report measures of mental health parameters including mood, anxiety, stress, and resilience both at baseline and post-intervention. Results were remarkable for statistically significant differences in measures of the students’ feelings of anger control, fatigue and inertia [126]. Of note, this is one of a few trials that compares the effects of yoga to that of another form of exercise (in this case, physical education as an exercise control group). More studies comparing the yoga with standard exercise are warranted as benefits can certainly be seen for both. 

Another interesting study from 2010 examined a school-based mindfulness and yoga intervention program in four inner city public schools in Baltimore, MD. Each of the schools was randomly assigned to undergo either a 12-week yoga/mindfulness intervention program or a wait-list control. Mental health parameters were also assessed in the trial including stress responses, feelings of depression, emotional arousal, control of thoughts and student social interactions. Positive outcomes were again seen in the intervention group compared to the controls in a number of these different psychological variables. Both of these studies are significant in that they suggest that mindfulness-based programs may be of great benefit for youths, especially in helping them to cope with daily stressors. In addition, both studies illustrate that a school-based mindfulness/yoga program is easily accessible, acceptable to students/staff and feasible to incorporate into schools that serve underprivileged and stressed youth [127]. 

A landmark study was recently performed in 2016, again in two inner-city public schools in Baltimore, MD. This large RCT examined in 300 students, randomized to receive either the Mindfulness Based Stress Reduction (MBSR) program, developed by Jon Kabat-Zinn, or a healthy education program (Healthy Topics (HT)) for an intervention period of 12 weeks. The two groups were comparable at baseline: average age was 12 years, 50.7% female, 99.7% African American, and 99% eligible for free lunch. After the 12-week intervention program, the results were quite significant with the MBSR students reporting lower levels of depression, self-hostility and other negative affective complaints than the group who received HT education (*p* < 0.05) [128]. These outcomes suggested that underprivileged, vulnerable and disadvantaged youths could significantly benefit from mindfulness-based programs in school, particularly with negative psychological symptoms and developing improved coping mechanisms for stress. 

Of great significance is the impact that these positive outcomes may have as children mature—it is a well-appreciated fact that many adult diseases have their genesis in childhood, especially those due to high exposure to stress and trauma [128]. In a research review from 2014, Hagen et al. proposed that educational facilities including pre-schools, schools, and community centers offer yoga and mindfulness-based programs for children and adolescents. The authors suggested that these school-based programs may help students improve self-regulation of emotions, stress-coping skills, resiliency and overall mood. They concluded that for young people to start practicing mind-body techniques from an early age may prevent future generations from experiencing more stress in their adult lives. [114].

## 14. Conclusions

Ongoing research into yoga and mindfulness-based practices continues to reveal and uncover health benefits, supporting its use in health management. 

Although there have been many published studies and research trials demonstrating and advocating yoga as a treatment and/or adjunct therapy for multiple disease entities, there are limitations that should be noted for a number of these studies. Many of these studies are single-center trials, have small and/or low-powered sample sizes and use non-standardized methodologies with short follow-up periods. It also should be noted that the field of yoga research encompasses the inherent dilemma of the wide variety of yogic practices used as interventional therapies. Larger, multi-centered studies using standardized yoga programs and uniform methodologies with long-term follow-up and outcomes are needed. 

The practice of yoga is not as easy or as quick as taking medication, but mounting evidence suggests it is worth the effort and investment. Yoga helps one to reconnect with oneself. It can help to uncover why and how one’s illness may have started, and can work with the body to start the recovery period from the ground up. The practice can help one to see how they may be reacting to the world around them, and may help them learn to respond from a different perspective. Slowing down, quieting our minds and connecting with our inner selves all help to bring one into the present moment. This can ultimately help to relieve one from the pressures and stressors from the hustle and bustle of this very busy world. 
